# Automatic Inattention to Attractive Alternative Partners Helps Male Heterosexual Chinese College Students Maintain Romantic Relationships

**DOI:** 10.3389/fpsyg.2019.01687

**Published:** 2019-07-18

**Authors:** Yidan Ma, Weifeng Xue, Shen Tu

**Affiliations:** ^1^Department of Psychology, Institute of Education Science, Leshan Normal University, Leshan, China; ^2^Applied Psychology, School of Public Administration, Guizhou University of Finance and Economics, Guiyang, China

**Keywords:** relationship maintenance, attractive alternatives, attentional bias, social cognition, romantic love

## Abstract

Heterosexual individuals may possess evolved psychological mechanisms that help protect their ongoing romantic relationships against external threats from other attractive individuals. The current study used love priming and a dot-probe task to examine the attentional bias associated with long-term relationship maintenance by comparing between 52 single heterosexual men and 57 heterosexual men in exclusive romantic relationships, in the Chinese context. The results showed that single men responded to love priming with greatly increased attention to and difficulty disengaging from attractive women, whereas committed men were largely inattentive to attractive alternatives irrespective of the situation. The present findings provide evidence on the domain of relationship maintenance from a Chinese cultural context, and suggest that Chinese men protect an ongoing relationship by being automatically inattentive in early-stage attentional processing to attractive women who could serve as attractive alternatives.

## Introduction

From the social psychology perspective, long-term pair-bonding can help satisfy individuals' fundamental needs for positive social bonds (Baumeister and Leary, 1995), and social evolutionary studies have found that long-term romantic relationship can provide strong family ties with both male and female sides of the family and bind different groups together (Chapais, 2013); a stable and satisfying long-term romantic relationship can also facilitate positive emotional experience and better personal well-being and physical health (Baumeister and Leary, 1995; Proulx et al., 2007; Robles et al., 2014). From the evolutionary perspective, the social bonds formed and maintained in romantic relationships can offer heavy investment and reproductive benefits, such as survival for both adults and offspring (Buss and Schmitt, 1993; Fletcher et al., 2015); and for heterosexual men, long-term romantic relationship can offer the possibility of monopolizing a woman's lifetime reproductive resources and development of long-term alliances with the woman's kin (Smuts, 1991, cited in Buss and Schmitt, 1993). Thus, committed individuals use many tactics to maintain their long-term romantic relationships, such as forgiveness, gratitude, positive illusions, and so on (Ogolsky et al., 2017). One of the primary ways that partners can maintain their relationships is by mitigating potential threats to their relationship from attractive others (Ogolsky et al., 2017), because interest in attractive alternatives is an important factor threatening ongoing romantic relationships that could cause infidelity and relationship dissolution (Miller, 1997; Le et al., 2010; McNulty et al., 2018). Infidelity can cause many damaging problems in relationships. For example, those who participate in acts of infidelity (as compared to faithful partners) experience greater levels of guilt and shame and report more psychological distress, such as greater depressive symptoms and lower general well-being (Fisher et al., 2008; Hall and Fincham, 2009), while the faithful partners suffer from disappointment, self-doubt, and anger (Buunk, 1995). Thus, heterosexual individuals appear to possess evolved psychological mechanisms to protect against relationship threats from physically attractive individuals (to the best of our knowledge, there is no similar research on homosexual individuals) (e.g., Maner et al., 2009; Ma et al., 2015). Committed individuals tend to derogate attractive opposite-sex persons to resist temptation from them. For example, committed individuals devalue the attractiveness of alternatives (e.g., Johnson and Rusbult, 1989; Simpson et al., 1990; Cole et al., 2016), memorize their faces less (Karremans et al., 2011; this study only focused on mated women), remember more negative behaviors engaged in by them (Visserman and Karremans, 2014), and are inattentive to them in early-stage visual processing when relationship motivation is activated (e.g., Maner et al., 2009). However, there is also some evidence that does not support the “derogation of alternatives” function to maintain long-term romantic relationships. For example, single women have equally poor memory for attractive men as mated women do (Wang et al., 2016); and mated individuals become sexually aroused in response to a scenario describing a casual sex mating opportunity with an attractive person, though less so than single individuals (Stone et al., 2011). On the other hand, for attractive same-sex persons who could serve as intrasexual rivals, committed women tend to show attentional adhesion to them in early-stage attentional processes, which could facilitate the identification of potential intrasexual threats to help guard their partners from infidelity (Maner et al., 2007a). For example, committed women who were primed with love that may trigger concerns about partner infidelity or those who felt insecure about their relationship could find it hard to disengage from attractive rivals (Maner et al., 2007a; Ma et al., 2015). Attentional bias associated with relationship maintenance is of great importance, as it could shape individuals' adaptive social cognition and actions (Maner et al., 2007a). The aim of this study was to explore whether mated male college students in the Chinese cultural context possess relationship maintenance mechanisms (e.g., inattention to attractive alternatives, attentional adhesion to intrasexual rivals) in early-stage attentional processing compared with single men, using a modified dot-probe paradigm, which can provide the baseline to identify attentional biases.

### Inattention to Attractive Alternatives and Relationship Maintenance

Previous researchers have used mating-related motivation priming, which closely linked to differential reproductive success can immediately impact individuals' perception of other people (Maner et al., 2007b; Kenrick et al., 2010), and the dot-probe task to explore committed individuals' attentional biases associated with romantic relationship maintenance in early-stage attentional processes. Some studies have found that committed individuals may reduce relationship threats from attractive alternatives by reducing attention to them when relationship motivation is activated (e.g., sexual priming, love priming) (Maner et al., 2008b, 2009; Zhang et al., 2017).

However, as some of the above results imply, there might be differences in this “inattention” between committed men and committed women after love priming. Maner et al. (2008b) asked participants to write a brief essay about a time when they felt a strong, happy love for their current partner (participants in the control condition wrote a brief essay about any happy time), and found that committed individuals of both sexes had significantly decreased attention to attractive alternatives; however, comparing reaction times (RTs) to attractive alternatives in the love priming condition to those in the control priming condition, this love priming effect was somewhat larger among men (a reduction in attention of 134 ms) than among women (a reduction in attention of 40 ms), though non-significantly. This lesser attentional decrease in committed women seems consistent with the established attentional biases of committed women in the Chinese cultural context—that is, committed women did not decrease attention toward attractive alternatives after love priming, which presented words associated with love to participants for 500 ms, because they were already relatively inattentive to attractive alternatives in the baseline (control priming) condition (Ma et al., 2015). Following the above findings, in the present study, we used the love priming procedure from Ma et al. (2015) to activate Chinese male mating-related motivation in order to examine whether committed men would show inattention to attractive alternatives regardless of the situation (love priming or control priming) or greatly decrease attention to attractive alternatives after love priming for relationship maintenance.

Historically, traditional Chinese culture places strict moral constraints on women's chastity and loyalty to their husbands, including refraining from premarital or extramarital sex and not remarrying after their husbands die (Li and Wei, 2010), while Chinese men were permitted to have (legally own) one wife and several concubines (Xu and Ocker, 2013). Influenced by traditional Chinese culture, “male-superior norms” are still prominent in China (Higgins et al., 2002); for instance, both men and women are more tolerant of male than female infidelity (Zheng et al., 2011). Indeed, the prevalence of infidelity (including commercial and non-commercial sex) among Chinese men (13.6%) is higher than that among Chinese women (4.2%) in modern society (Zhang et al., 2012). Meanwhile, to link up with other findings the evidence that male (vs. female) college students judge infidelity to be more morally acceptable (Xiao et al., 2017), we first predicted that committed men would show difficulty disengaging from attractive women before love priming. Second, romantic love is typically defined as “the constellation of behaviors, cognitions, and emotions associated with a desire to enter or maintain a close relationship with a specific other person” (Aron and Aron, 1991, cited in Diamond and Dickenson, 2012). Thus, for mated individuals, romantic love functions as a commitment device that can promote long-term commitment, sustain long-term bonds (Gonzaga et al., 2006) and arouse relationship motivation, which could help to reduce attention to attractive alternatives (Maner et al., 2008b). For single individuals, on the other hand, feeling of romantic love could arouse their mating motivation to seek a partner to enter a close relationship, and lead them to be more attentive to cues of female facial beauty (Buss and Schmitt, 1993; Kenrick et al., 2010; van Hooff et al., 2010). Thus, we predicted that committed men in our study would reduce attention to attractive alternatives after love priming at early-stage attentional processes, while single men would increase attention toward and disengage with greater difficulty from attractive women in such a context.

### Attentional Adhesion to Intrasexual Rivals and Relationship Maintenance

Previous studies have found that committed women's mate-guarding motivation made it difficult for them to disengage from attractive women (e.g., Ma et al., 2015), who served as intrasexual rivals and threats to their relationship and reproductive success (Buss and Shackelford, 1997). Attentional adhesion to attractive women may help committed women identify and evaluate potential rivals, and serve to protect their current relationship (e.g., Maner et al., 2007a).

Meanwhile, evolutionary theories of mating suggest that women in long-term relationships tend to seek highly attractive men from extra-pair relationships to gain good genes (Pillsworth and Haselton, 2006a), especially near ovulation (Pillsworth and Haselton, 2006b). It seems that attractive men can also serve as rivals to committed men, who show mate-guarding behaviors (e.g., tracking cues of partner's attraction to other men) to maintain their relationship under perceived threats from intrasexual rivals (Haselton and Gangestad, 2006). Thus, the third question we wanted to probe is whether Chinese men will increase attention toward attractive rivals when primed with love, which could help relationship maintenance, as in the committed Chinese women in Ma et al. (2015). Given that women's fidelity is highly valued in Chinese culture (Li and Wei, 2010) and that men tend to prefer faithful women in a long-term romantic relationship (Buss and Schmitt, 1993), Chinese men may be less likely to doubt their mates' fidelity, and thus committed men might not increase their attention toward attractive rivals after love priming.

To sum up, we will test five hypotheses in this study as follows:

- Hypothesis 1: Compared to RTs toward neutral picture pairs (see Data Preparation section), committed (heterosexual) men will show difficulty disengaging from attractive women in the control priming condition.
- Hypothesis 2: Compared to committed men in the control priming condition, committed men will reduce attention to attractive alternatives in the love priming condition.
- Hypothesis 3: Compared to single men in the control priming condition, single men will increase attention toward attractive women in the love priming condition; and compared to RTs toward neutral pictures pairs, single men will disengage with greater difficulty from attractive women in the love priming condition.
- Hypothesis 4: Committed men will be less attentive than single men to attractive women in the love priming condition.
- Hypothesis 5: Compared to committed men in the control priming condition, committed men will not increase attention toward attractive men in the love priming condition.

## Methods

### Participants

One hundred and nine male undergraduates (age range = 18–25 years) participated in the study in exchange for course credit. All reported being heterosexual, were right-handed, and had normal or corrected-to-normal vision. Of the participants, 52 were single (mean age = 20.87 ± 1.43) and 57 were currently in a committed, exclusive romantic relationship (mean age = 21.44 ± 1.74; mean relationship length = 18.18 ± 10.22 months [length range = 5–48]). The committed participants completed the commitment subscale of the Companionate Love Scale (Hatfield and Rapson, 1996), which comprises four items rated from 1 (not at all true) to 9 (definitely true) to measure commitment (α = 0.71). A sample item is “I am committed to maintaining my relationship with my girlfriend.” On average, these participants were moderately or highly committed to their current partner (*M* = 7.57, *SD* = 0.88). Ethical approval for this study was obtained from the local research ethics committee, and informed consent was obtained from all participants.

### Materials

#### Priming Words

The task included 15 love words and 15 control words. First, we administered the 20 love words, which were also used in Ma et al. (2015), to 30 male undergraduates (mean age = 20.78 years, *SD* = 1.83; none of whom was involved in other parts of this study) to rate the love words in terms of the extent to which they were associated with love (1 = not at all to 7 = completely) and with two emotional dimensions: pleasure (1 = very displeasing to 9 = very pleasing) and excitement (1 = very calm to 9 = very exciting). Then we picked up the top 15 words (see Supplementary Material) based on love score (love: *M* = 6.25, *SD* = 0.20; pleasure: *M* = 7.07, *SD* = 0.70; excitement: *M* = 5.31, *SD* = 0.69). Fifteen control words were taken from the Chinese Affective Words System (Wang et al., 2008) to match the priming words in terms of pleasure, *M* = 7.00, *SD* = 0.16, *t*_(15)_ = −0.36, *p* = 0.722, and excitement, *M* = 5.59, *SD* = 0.30, *t*_(19)_ = 1.40, *p* = 0.18.

#### Stimulus Materials

All the stimuli materials had been validated in Ma et al. (2015). The facial photographs used were of unfamiliar college-age Chinese people with a neutral expression and were edited to grayscale and balanced for size, brightness, and contrast using Adobe Photoshop software. All the faces were pre-rated for physical attractiveness (1 = very unattractive to 7 = very attractive) by 30 undergraduate judges (12 males and 18 females, mean age = 20.73 years, *SD* = 1.55) (Ma et al., 2015). This study included four facial types: (a) 20 highly attractive men (*M* = 5.38, *SD* = 0.87), (b) 20 highly attractive women (*M* = 5.45, *SD* = 0.87), (c) 20 average-looking men (*M* = 3.17, *SD* = 0.62), and (d) 20 average-looking women (*M* = 3.20, *SD* = 0.60). In addition, 120 neutral pictures of household furniture (e.g., table, chair, sofa) were edited to match the facial stimuli in size, brightness, contrast, and color; of these neutral pictures, 20 were used in the practice trials and the remaining 100 were used in the experimental trials.

### Design and Procedure

**Figure 1:**
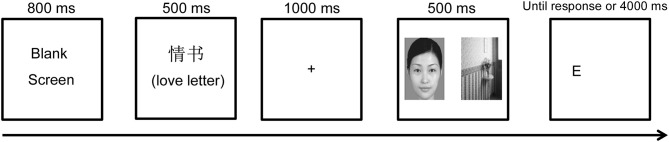
Example of the dot probe task paradigm procedure (cited in Ma et al., 2015).

Participants were told that the experiment investigated cognitive ability, and then performed the dot-probe task used in Ma et al. (2015) to assess attentional bias. Each trial began with a blank screen displayed for 800 ms, followed by a priming (or control) word displayed on the center of the computer screen for 500 ms. After the word disappeared, a black fixation cross was displayed at the center for 1,000 ms and was subsequently replaced by a pair of pictures (either a face-neutral or a neutral-neutral pair) displayed for 500 ms. Immediately after the offset of each picture pair, a probe target (“E” or “F”) was presented at the location of one of the pictures and the participant had to indicate the type of probe as quickly as they could. The probe was displayed until a response was made or up to a maximum of 4,000 ms (see Figure 1). Participants completed 10 practice trials and four blocks that each had 90 experimental trials. Each picture was presented only once in each block, and each pair was matched in a random order, with no pair repeated. The types and locations of the probes were counterbalanced across the experiment. After the experiments, committed participants completed a questionnaire measuring age and relationship length, as well as the commitment subscale of the Companionate Love Scale, to assess their commitment to their current relationship; all participants were carefully probed for suspicion, and none recognized the true purpose of the experiment.

### Data Preparation

Trials with RTs of <200 ms and more than three standard deviations above the sample mean and those with errors were excluded from the analyses. Following Maner et al. (2008b) and Ma et al. (2015), this study only focused on the disengagement component of attention and all data analyses were based on the disengagement indices of the RTs. The disengagement indices could control for overall group differences (Ellenbogen and Schwartzman, 2009); for example, in this study, the RTs of all target types in the love priming condition were faster than RTs in the control priming condition, but the index scores could exclude overall group differences to show the pure priming effect. The disengagement indices were calculated by subtracting RTs for trials with neutral-neutral picture pairs from RTs for incongruent trials, which had face-neutral picture pairs. A positive score on the disengagement index that is significantly different from zero is indicative of difficulty with disengagement from facial pictures compared with neutral pictures. A negative score significantly different from zero suggests faster attending away from facial pictures as compared to neutral pictures. A score of zero implies no differences in attentional disengagement for neutral vs. facial pictures (Koster et al., 2006; Salemink et al., 2007).

For a planned contrast among attractive opposite-sex, attractive same-sex, and average-looking targets, the mean disengagement indices for average-looking men and women were calculated. To ensure that using mean disengagement indices of average-looking men and women was feasible, we conducted a 2 (relationship status: single man vs. committed man) × 2 (priming condition: love priming vs. control priming) × 2 (target type: average male target vs. average female target) repeated-measures analysis of variance (ANOVA), and found that there were no differences between the two target types (all *p*s > 0.402). Further, a 2 (relationship status: single man vs. committed man, between-subject) × 2 (priming condition: love priming vs. control priming, between-subject) × 3 (target type: attractive male vs. attractive female vs. average-looking targets, within-subject) repeated-measures ANOVA was performed. Only the main effects and interactions relevant to the study's hypotheses are reported.

## Results

**Figure 2:**
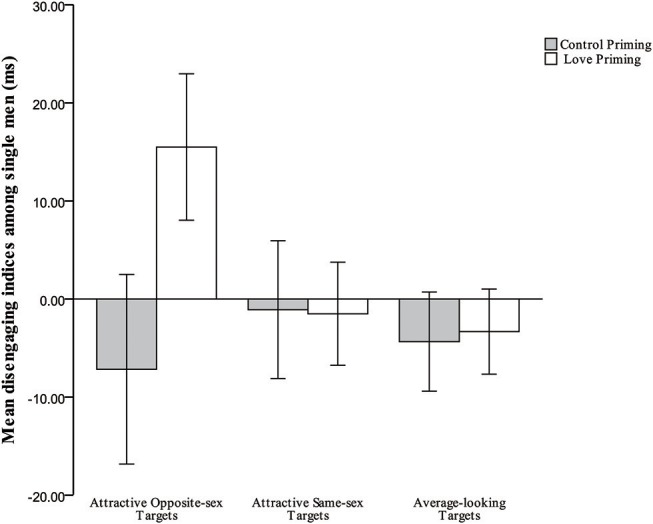
Mean indices of disengagement from all target types for single men in the love priming and control priming conditions.

**Figure 3:**
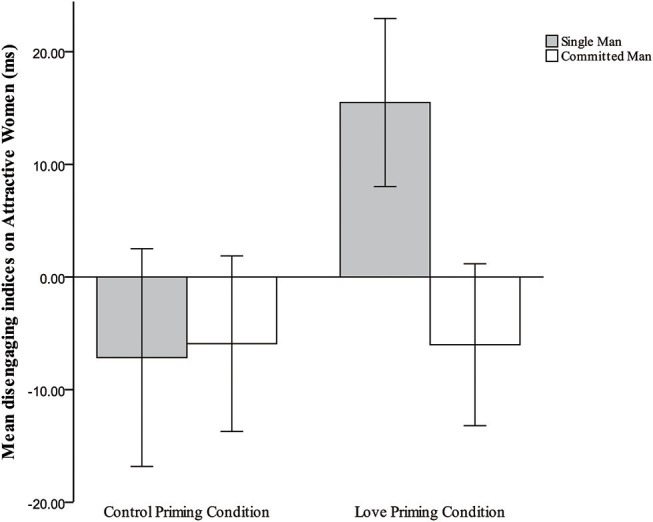
Mean indices of disengagement from attractive women for single and committed men in the love priming and control priming conditions.

**Figure 4:**
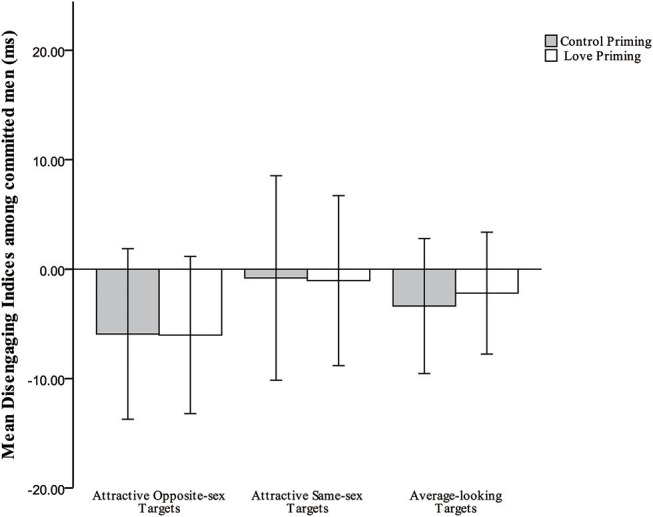
Mean indices of disengagement from all target types for committed men in the love priming and control priming conditions.

Disengagement indices by target type, priming condition, and relationship status are presented in Table 1. The three-way interaction from the 2 × 2 × 3 mixed-model ANOVA was significant, *F*_(2, 210)_ = 6.842, *p* = 0.001, partial η^2^ = 0.061. Additional simple-effects tests were performed to examine hypothesis 2 (compared to committed men in the control priming condition, committed men would reduce attention to attractive alternatives in the love priming condition), and hypothesis 3 (compared to single men in the control priming condition, single men would increase attention toward attractive women in the love priming condition), and hypothesis 4 (committed men would be less attentive than single men to attractive women in the love priming condition). For single men, compared to the baseline condition, love priming increased their attention only to attractive women, *F*_(1, 105)_ = 15.284, *p* < 0.001, partial η^2^ = 0.127 (see Figure 2), while among committed men, no significant effect of priming was observed for attentional biases toward attractive women, *F*_(1, 105)_ = 0.000, *p* = 0.986; in addition, committed men were significantly less attentive than were single men to attractive women in the love priming condition, *F*_(1, 105)_ = 14.563, *p* < 0.001, partial η^2^ = 0.122 (see Figure 3). Those results support the hypothesis on single men, but partially on committed men. Hypothesis 5 was that compared to committed men in the control priming condition, committed men would not increase attention toward attractive rivals in the love priming condition. Consistent with the hypothesis, result showed no significant effect of priming for attentional biases toward attractive men among committed men, *F*_(1, 105)_ = 0.002, *p* = 0.963 (see Figure 4). No other significant effect was observed under the baseline condition or love priming condition (all *p*s > 0.122).

**Table 1:** Mean RT (ms), disengagement indices and SD by target type, priming condition, and relationship status.

**Target type**	**RT type**	**Love priming condition**		**Control priming condition**	
		**Single (*n* = 27)**	**Committed (*n* = 28)**	**Single (*n* = 25)**	**Committed (*n* = 29)**
N-N		547 (58)	567 (67)	582 (39)	588 (65)
Attractive men-N	Incongruence RT	546 (60)	566 (65)	580 (34)	587 (72)
	Disengagement	−2 (14)	−1 (21)	−1 (18)	−1 (25)
Average men-N	Incongruence RT	542 (54)	565 (68)	578 (37)	584 (72)
	Disengagement	−5 (19)	−2 (18)	−4 (17)	−4 (21)
Attractive women-N	Incongruence RT	563 (64)	561 (67)	575 (41)	582 (68)
	Disengagement	15 (19)	−6 (19)	−7 (24)	−6 (21)
Average women-N	Incongruence RT	545 (57)	565 (65)	577 (42)	585 (71)
	Disengagement	−2 (16)	−3 (16)	−5 (13)	−3 (18)

*RT, reaction time; N, neutral picture; standard deviations are shown in parentheses*.

To explore whether the commitment and relationship length would affect the results, we conducted further analysis using only committed men's data, specifically, a 2 (priming condition: love priming vs. control priming) × 3 (target type: attractive male vs. attractive female vs. average-looking targets) repeated-measures ANOVA including logged relationship length, Companionate Love Scale score as covariates, the three-way interaction effect was not significant, *F*_(2, 106)_ = 0.007, *p* = 0.993, no other significant effects were observed (all *ps* > 0.699), and the same 2 (priming condition) × 3 (target type) repeated-measures ANOVA without logged relationship length and committed scores as covariates, the three-way interaction effect was also not significant, *F*_(2, 110)_ = 0.042, *p* = 0.958, no other significant effects were observed (all *ps* > 0.169). The results showed that those covariates would not affect the results in this study.

To test hypothesis 3 (compared to RTs toward neutral picture pairs, single men would disengage with greater difficulty from attractive women in the love priming condition), and hypothesis 1 (compared to RTs toward neutral picture pairs, committed men would show difficulty disengaging from attractive women in the control priming condition), we conducted the independent-samples *t*-test to compare disengagement indices with zero. The results confirmed that the disengagement index of single men for attractive opposite-sex persons was significantly >0 under the love priming condition, *t*_(26)_ = 4.152, *p* < 0.001, indicating that single men had difficulty in disengaging attention from attractive women after love priming. Among committed men, the disengagement index for attractive women was not significantly different from zero either in the baseline, *t*_(28)_ = −1.519, *p* = 0.140, or in the love priming condition, *t*_(27)_ = −1.675, *p* = 0.105, indicating that committed men had no difficulty in disengaging attention from attractive women regardless of the condition. Furthermore, the disengagement index of committed men for attractive same-sex persons was not significantly different from zero after love priming, *t*_(27)_ = −0.269, *p* = 0.790, nor were there any significant differences between zero and other disengagement indices (all *p*s > 0.133).

## Discussion

In the current study, we explored automatic attentional biases associated with long-term romantic relationship maintenance among heterosexual male college students in the Chinese cultural context. The results showed that when mental representations associated with love were activated, single men increased their attention toward and had difficulty in disengaging from attractive women who could be potential mates, whereas committed men were inattentive to attractive opposite-sex persons who could serve as attractive alternatives and threaten their ongoing relationship under both the baseline and love priming conditions. Moreover, committed men did not show increased attention to attractive same-sex persons, who could be intrasexual rivals, in the love priming condition.

In this study, feelings of romantic love may have activated single men's motivation to seek an attractive partner and thus increased attention only toward and resulted in difficulty disengaging from attractive women. This result is consistent with the evolutionary perspective, in which men tend to place a premium on the facial attractiveness of potential mates (Buss and Schmitt, 1993), which can predict health and reproductive success (Jones et al., 2001; Pflüger et al., 2012). Conversely, in accordance with previous evidence that romantic love can help people suppress thoughts of and to reduce interest in attractive alternatives (Gonzaga et al., 2008; Maner et al., 2008b), the committed men in this study did not increase attention to attractive women as single men did, but instead were as inattentive to them as in the baseline condition after love priming, which may activate committed men's motivation for relationship maintenance. The above results are consistent with the evolutionary perspective, in which fundamental social motives may immediately impact individuals' perception and behaviors, which could increase reproductive fitness (Kenrick et al., 2010).

However, in contrast to our hypothesis, committed men did not greatly reduce their attention to attractive alternatives in the love priming condition because they were already inattentive to attractive women in the baseline condition. In addition, the results were those of inconsistent with Maner et al. (2008b), where committed individuals, especially committed men, significantly reduced their attention to attractive opposite-sex individuals after love priming. One possibility for the different results could be the difference in attention to attractive alternatives in the baseline condition between samples from different cultures. In this study, we used disengagement indices, which can directly present participants' attentional biases toward all target types by comparing RTs for face-neutral trials to those for neutral-neutral trials; we found that committed Chinese men were inattentive to attractive alternatives in the baseline condition. This result might be because the committed men in this study were not attracted to alternatives, and, therefore, did not have to reduce their attention in order to resist temptation from attractive alternatives after love priming. But as Maner et al. (2008b) did not provide baseline RTs toward attractive alternatives under a control priming condition among committed individuals, analogous to the disengagement indices in this study, we cannot directly compare the two. However, according to the findings from previous studies in Western cultures showing that committed men continued to be interested in attractive women and continued looking for better alternatives until the motivation for relationship maintenance was activated (Maner et al., 2007a; Mitrovic et al., 2018) and that physically attractive alternatives were particularly threatening to men's relationships (Kenrick et al., 1989; Plant et al., 2010), attractive alternatives may be perceived as a strong threat by committed men in Western cultures when relationship maintenance motives are activated. Thus, the male participants in Maner et al. (2008b) might have needed to greatly reduce attention to attractive alternatives in order to resist this threat under the love priming condition. Another possibility could be differences in primes between the studies. Love priming in Maner et al. (2008b) involved asking participants to write a brief essay about a time when they felt a happy love for their current partner, while in the present study, love priming words were presented for 500 ms to participants. It seems that the love priming effects might have been less strong in our study (i.e., under the latter prime), and therefore that participants did not show strong avoidance motivation toward attractive alternatives or greatly reduce attention to them.

Committed Chinese women in Ma et al. (2015) increased their attention and found it hard to disengage from attractive same-sex individuals after love priming; this might be due to the love priming activating negative mental representations associated with love (e.g., infidelity) and thus triggering the mate-guarding motive, which could lead committed women to maintain attention to attractive same-sex individuals (Ma et al., 2015). Inconsistent with the findings in Ma et al. (2015), however, the committed Chinese men in this study, which used the same priming procedure as Ma et al. (2015), did not increase attention to attractive same-sex persons in the love priming condition. There are several possibilities that may account for this discrepancy: (1) Committed women tend to compete with physically attractive women for mate retention (Fink et al., 2014), while dominant men (e.g., high social prestige) may be stronger intrasexual rivals for committed men (Maner et al., 2008a). However, both Ma et al. (2015) and this study used only facial attractiveness to explore the relationship maintenance mechanism. (2) Men and women may have developed different strategies to maintain relationships: women tend to focus their attention on potential rivals whereas men show increased sensitivity to their own partners when threatened by partner infidelity (Ein-Dor et al., 2015). (3) The identification of and sensitivity to potential rivals seems more important to committed women than to committed men. This may be because, first, in Chinese culture, women's fidelity is highly valued (Li and Wei, 2010), including when choosing long-term romantic partners (Buss and Schmitt, 1993), and therefore, men may be less likely to doubt their mates' fidelity. Conversely, because of the division of social roles [e.g., men more often earn more money, while women more often do more housework and childcare and rely on their partner for financial resources (Zhang et al., 2014)], men in relationships might have had more exposure than women to precursors to infidelity and might be more responsive to those precursors (Zhang et al., 2012). Thus, committed men may have a greater probability of infidelity compared to committed women. Second, women are more likely than are men to lose romantic partners to attractive same-sex rivals (Schmitt, 2004), and thus, intrasexual rivals may be stronger threats to committed women than to committed men. (4) In China, men more easily perceive love as romantic than do women (Yin et al., 2013), and heterosexual men in love perceive their relationships as being better than their partners do (Zhou et al., 2017); women instead tend to associate love in general with jealousy and betrayal (Zang, 2013) and feel stronger romantic jealousy compared to men (Chen, 2010). Thus, love priming could more easily trigger concerns about partner infidelity and jealousy in women than in men, which might activate a mate-guarding motive and lead the committed women to increase and hold their attention to attractive same-sex individuals (Li et al., 2015; Ma et al., 2015).

However, there are several limitations to our study. First, the study participants were unmarried college student. Thus, we may not be able to generalize our conclusions to committed men who are already married. Our future studies will explore the relationship maintenance mechanism of individuals who are married or have children with their partner. Second, the only target trait we chose to study was general facial attractiveness; that is, we overlooked facial traits such as width-to-height ratios, which are positively associated with perceived social dominance (Valentine et al., 2014), and social dominance traits, such as social prestige and financial success. Because women tend to give more weight to social dominance than to physical attractiveness in mating-related evaluations of men (Maner et al., 2008a) and men feel more jealousy when rivals are high in the trait of dominance (Dijkstra and Buunk, 2002), dominant men may serve as strong rivals to committed men. Thus, future research should investigate committed men's attentional bias associated with relationship maintenance in the face of dominant men. Last, we ignored perceived power, which could influence individuals' attentional biases toward attractive opposite-sex individuals, because power increases confidence in the ability to attract partners (Lammers et al., 2011) and is positively associated with infidelity. To link this up with Chinese culture, in which “male-superior norms” are still prominent, perceived power may play an important role in attentional biases associated with relationship maintenance and need future research to further explore.

## Conclusion

The present findings provide evidence from a Chinese cultural context in the domain of relationship maintenance and show that Chinese men protected an ongoing relationship by being inattentive to attractive alternatives in early-stage attentional processes.

## Data Availability

The datasets for this manuscript are not publicly available because all relevant data is contained within the manuscript and the raw data file is available from the corresponding authors. Requests to access the datasets should be directed to mayidan114@qq.com.

## Author Contributions

YM conceived and designed the experiments. WX analyzed the data. YM and ST programmed experimental procedures and made experimental materials. YM and WX wrote the paper.

### Conflict of Interest Statement

The authors declare that the research was conducted in the absence of any commercial or financial relationships that could be construed as a potential conflict of interest.
