# Supplementary material for: Automatic Inattention to Attractive Alternative Partners Helps Male Heterosexual Chinese College Students Maintain Romantic Relationships
Source: Front Psychol. 2019 Jul 18;10:1687. doi: 10.3389/fpsyg.2019.01687 (PMC6657529; doi:10.3389/fpsyg.2019.01687)
Supplement: Supplementary file 1 [file Table_1.DOCX]

Priming words:

执子之手 [love you from the depths of my heart] 婚纱 [wedding veil]

与子偕老 [accompany you to the end of my life] 恋人 [lovers]

两情相悦 [resonance between two lovers] 情侣 [sweethearts]

比翼双飞 [go with each other all the time as lovers] 蜜月 [honeymoon]

一见钟情 [fall in love at first sight] 鸳鸯 [mandarin duck (symbol of love) ]

浓情蜜意 [great tenderness between lovers] 婚戒 [wedding ring]

如胶似漆 [love each other dearly] 情书 [love letter]

情人节 [Valentine’s Day]

Control words:

欢乐 [happy] 欢快 [bright] 高尚 [noble] 新奇 [newfangled]

渊博 [profound] 欢喜 [joyful] 愉快 [pleasant] 辉煌 [splendent]

精彩 [wonderful] 吉利 [lucky] 兴奋 [excited] 狂欢 [orgiastic]

壮丽 [gallant] 自豪 [proud] 珍贵 [precious]
